# Substantial Sparing of Organs at Risk with Modern Proton Therapy in Lung Cancer, but Altered Breathing Patterns Can Jeopardize Target Coverage

**DOI:** 10.3390/cancers14061365

**Published:** 2022-03-08

**Authors:** Camilla Grindeland Boer, Kristine Fjellanger, Inger Marie Sandvik, Maren Ugland, Grete May Engeseth, Liv Bolstad Hysing

**Affiliations:** 1 Department of Oncology and Medical Physics, Haukeland University Hospital, 5021 Bergen, Norway; camilla.grindeland.boer@helse-bergen.no (C.G.B.); inger.marie.sandvik@helse-bergen.no (I.M.S.); maren.ugland@helse-bergen.no (M.U.); grete.may.engeseth@helse-bergen.no (G.M.E.); liv.bolstad.hysing@helse-bergen.no (L.B.H.); 2 Institute of Physics and Technology, University of Bergen, 5007 Bergen, Norway; 3 Department of Clinical Science, University of Bergen, 5021 Bergen, Norway

**Keywords:** locally advanced non-small cell lung cancer, NSCLC, pencil beam scanning proton therapy, robustness, toxicity, breathing motion

## Abstract

**Simple Summary:**

Treatment of locally advanced non-small cell lung cancer (LA-NSCLC) is a fine balance between toxicity and cure. Modern proton therapy might offer a more gentle radiation treatment compared to state-of-the-art photon radiotherapy, but is also more susceptible to the influence of breathing motion and anatomical changes. In this study, the influence of such uncertainties on treatment delivery was thoroughly investigated. Modern proton therapy did indeed show potential to reduce the risk of toxicity for the heart and lungs. This potential was maintained under the influence of anatomical and delivery uncertainties. However, changes in breathing motion jeopardized the target dose distribution in a subset of patients. We therefore recommend imaging at onset or early in treatment to recognize these patients and adapt the treatment.

**Abstract:**

Enhancing treatment of locally advanced non-small cell lung cancer (LA-NSCLC) by using pencil beam scanning proton therapy (PBS-PT) is attractive, but little knowledge exists on the effects of uncertainties occurring between the planning (Plan) and the start of treatment (Start). In this prospective simulation study, we investigated the clinical potential for PBS-PT under the influence of such uncertainties. Imaging with 4DCT at Plan and Start was carried out for 15 patients that received state-of-the-art intensity-modulated radiotherapy (IMRT). Three PBS-PT plans were created per patient: 3D robust single-field uniform dose (SFUD), 3D robust intensity-modulated proton therapy (IMPT), and 4D robust IMPT (4DIMPT). These were exposed to setup and range uncertainties and breathing motion at Plan, and changes in breathing motion and anatomy at Start. Target coverage and dose-volume parameters relevant for toxicity were compared. The organ at risk sparing at Plan was greatest with IMPT, followed by 4DIMPT, SFUD and IMRT, and persisted at Start. All plans met the preset criteria for target robustness at Plan. At Start, three patients had a lack of CTV coverage with PBS-PT. In conclusion, the clinical potential for heart and lung toxicity reduction with PBS-PT was substantial and persistent. Altered breathing patterns between Plan and Start jeopardized target coverage for all PBS-PT techniques.

## 1. Introduction

State-of-the-art treatment for inoperable locally advanced non-small cell lung cancer (LA-NSCLC) is concurrent chemotherapy and intensity-modulated photon radiotherapy (IMRT) to a dose of 60 Gy. Still, 5-year survival rates for stage III disease are only around 30%, and side effects from treatment are common and potentially fatal, limiting the possibility for dose escalation with IMRT [1,2].

Proton therapy (PT) has advantageous depth dose characteristics with the potential to reduce side effects and facilitate dose escalation in LA-NSCLC patients [3,4,5,6,7]. Although phase II clinical trials have been promising [8,9,10], PT showed no advantage over IMRT in a randomized trial by Liao and colleagues [11,12]. These early clinical trials have mainly applied passive scattering PT, but state-of-the-art PT uses pencil beam scanning (PBS), allowing more conformal dose distributions with lower doses to critical organs [3,13].

PBS-PT is, however, not straightforwardly delivered in the thoracic region due to the inherent sensitivity to uncertainties [14,15]. Much concern has been dedicated to how the breathing motion of the primary tumor can interplay with PBS-PT spot delivery [16,17]. PBS-PT has therefore mainly been offered to patients with limited breathing motion [14]. However, recent studies with PBS-PT confirm that interplay uncertainties are canceled out by fractionation—as for IMRT—and more attention should be focused on changes in breathing patterns and anatomy [15,18,19,20,21].

Various optimization techniques for PBS-PT exist, and it is believed that these are differently influenced by uncertainties [14,16]. With single-field uniform dose (SFUD), each field delivers a uniform dose to the entire target volume, while intensity-modulated proton therapy (IMPT) contains non-uniform dose distributions for the individual fields. 3D and 4D robust optimization can be applied to account for uncertainties due to patient setup and proton range as well as breathing motion, respectively [22,23,24].

In theory, IMPT has the potential to produce the most conformal treatment plans, while SFUD and 4D robust optimization are strategies to increase robustness [17]. There is, however, limited knowledge on how different robustly optimized PBS-PT techniques perform in practice for LA-NSCLC patients, as the planning CT is commonly used for both optimization and evaluation [16]. We see a need to investigate this in order to guide the use of PBS-PT, balancing organ at risk (OAR) sparing and robustness for both targets and OARs. Furthermore, little knowledge exists on the dosimetric advantages of PBS-PT compared to state-of-the-art IMRT at the start of treatment. Comparisons for, e.g., patient selection between protons and photons are also usually carried out on the planning scan, even though it is known that robustness towards changes matters [25]. A few studies have focused on the impact of anatomical changes occurring during the six weeks of treatment that should be handled by means of adaptive radiotherapy (ART) [26,27,28].

The purpose of this prospective simulation study was to compare 3D robust SFUD, 3D robust IMPT and 4D robust IMPT in terms of target coverage and OAR sparing under the influence of setup and range uncertainties, breathing motion and interplay at planning, as well as changes in the breathing motion pattern and anatomy from the planning to the start of treatment. Further, using the clinical IMRT plan as a reference, our objective was to evaluate if the potential for OAR sparing expected at planning was persistent at the start of treatment.

## 2. Materials and Methods

### 2.1. Patient Material and Clinical IMRT Planning

Fifteen consecutive patients with stage III NSCLC receiving radiochemotherapy with curative intent at Haukeland University Hospital in Bergen, Norway, in 2019–2020 were prospectively included in an in silico simulation study. All patients gave informed consent, and the study was approved by the regional committee for medical and health research ethics (protocol code 2019/749).

Imaging was performed on a Big Bore CT scanner (Philips Healthcare, Best, The Netherlands), using a Posirest-2 support device (Civco Radiotherapy, Coralville, IA, USA) for fixation in the supine position with arms resting above the head, and the Philips bellows device for registration of the breathing curve.

4DCTs with 10 respiratory phases and deep inspiration breath hold (DIBH) CTs were acquired at planning (Plan) and at the start of treatment (fraction 2 or 3; Start). The average intensity projection (AIP) of the 4DCT was used for delineation and treatment planning. Gross tumor volumes (GTVs) for the primary tumor and lymph nodes were defined on the AIP, based on a diagnostic CT with intravenous contrast, an FDG-PET-CT, and biopsy of mediastinal lymph nodes. To define the internal GTVs (IGTVs), each 4DCT phase was blended with the AIP, and the structure was expanded to include the GTV positions on all phases. Exceptions from this were three patients treated in DIBH due to lung dose exceeding the constraints or large tumor motion blurring the 4DCT. In these cases, IGTV delineation included the GTV on three consecutive planning DIBH scans. A clinical target volume (CTV) was created using a 5 mm margin from the IGTV, without extending into uninvolved organs such as bone, heart, esophagus and major vessels. The GTVs and CTV were deformably mapped to all phases of the 4DCT and later used in 4DIMPT optimization. For clinical IMRT planning, a planning target volume (PTV) with 5 mm margin from the CTV was used. Target delineation was performed by the same oncologist (I.M.S.) on all Plan and Start scans. The lungs, heart, esophagus, spinal canal and, if relevant, the brachial plexus were delineated according to RTOG guidelines [29].

Clinical treatment planning was performed in Eclipse v. 15.6 (Varian Medical Systems, Palo Alto, CA, USA). All patients received IMRT with a prescribed dose of 60 or 66 Gy in 2 Gy fractions, depending on lung function, lung dose and proximity of the brachial plexus to the PTV. The beam configuration was adjusted to fit the anatomy of each patient, mainly using six beams and avoiding entry through the contralateral lung. For the PTV, D_98%_ > 95% of prescribed dose was required, and the maximum dose in the plan should be <107%. Dose constraints for OARs are listed in Table A1. The Acuros External Beam algorithm was used for dose calculation, and the plans were normalized to the median dose in the PTV.

The motion amplitude of the primary tumor at Plan and Start was evaluated in Eclipse, using deformable mapping of the primary tumor GTV from the AIP to each breathing phase of the 4DCT and measuring the motion of the GTV center of mass in all directions.

The AIP of the 4DCT acquired at Start was rigidly matched to the AIP at Plan using six degrees of freedom and a volume of interest covering the PTV, as well as skeletal structures and the body contour in proximity to the PTV.

### 2.2. Proton Therapy Planning

Proton planning was performed in RayStation v. 8B (RaySearch Laboratories, Stockholm, Sweden). To ensure high plan quality, all plans were made by an experienced planning expert within photon therapy and comparative proton planning (C.G.B.) and reviewed by an experienced medical physicist (M.U.). The 4DCT phases were deformably registered to their respective AIP, and the deformed target volumes (GTVs and CTVs) and OARs were mapped onto each phase. For the AIP scan, a density override representative for tumor tissue (1.06 g/cm^3^, ~40 HU) was used for all plans for the IGTV. For the 4DCT phases, the original density values were applied (i.e., no density override).

For each patient, three PBS-PT treatment plans were created on the Plan AIP using different optimization techniques: SFUD, 4DIMPT and IMPT. 3D robust optimization according to the minimax approach with setup uncertainty of 5 mm in each direction and 3.5% range uncertainty (21 scenarios) was used for SFUD and IMPT [22]. 4D robust optimization, applying the same settings for setup and range uncertainty on all 4DCT breathing phases (231 scenarios), was used for 4DIMPT [23]. In 3D and 4D robust optimization, the reference plan is evaluated in each uncertainty scenario, and in each iteration, the scenario with the currently worst objective value is improved. 3D and 4D robust optimization were applied for the CTV, and for the spinal canal if close to the CTV. Rescanning methods were not used.

**Figure A1:**
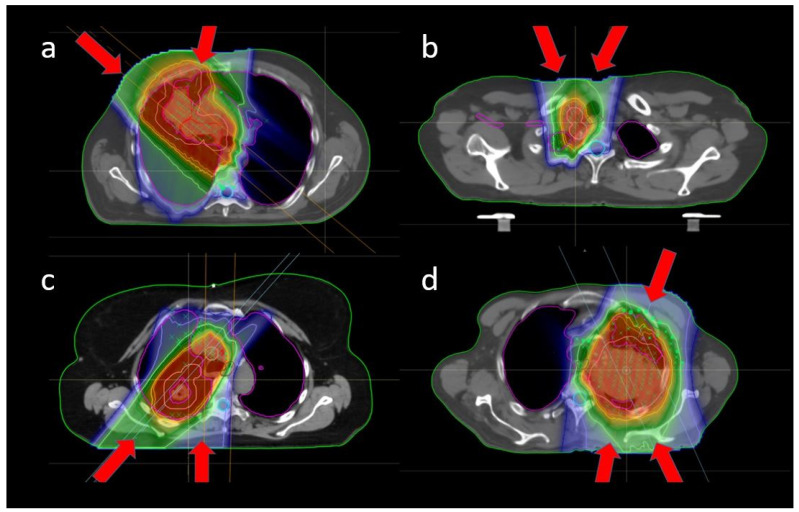
Common beam configurations for different tumor locations: (**a**) frontal oblique tumor with oblique and frontal fields, (**b**) nodes in the frontal part of the mediastinum with two slightly frontal oblique fields, (**c**) dorsal tumor with dorsal oblique and dorsal fields, and (**d**) large tumor near the brachial plexus, with slightly frontal oblique, dorsal and dorsal oblique fields.

Each plan had two (10 patients) or three (5 patients) coplanar fields with gantry angles carefully selected with regard to the patient anatomy and the distance between the beam entry and the CTV (Figure A1). For each patient, beam angles were individually selected, and the same field setup was used in the three PT plans. Range shifters of 4 cm or 7.5 cm were used for all fields, and the air gaps were 5–12 cm from the body contour depending on beam angles and risk of collision. Sigma of spot sizes in air at isocenter (without range shifter) were 3.7 to 7.2 mm depending on energy.

The same prescription as in the clinical plan was used, applying a relative biological effectiveness of 1.1 for protons. A generic IBA beam model was used for planning, and a Monte Carlo algorithm was used for dose calculation (using 0.5% statistical uncertainty).

### 2.3. Robustness Evaluation

**Figure 1:**
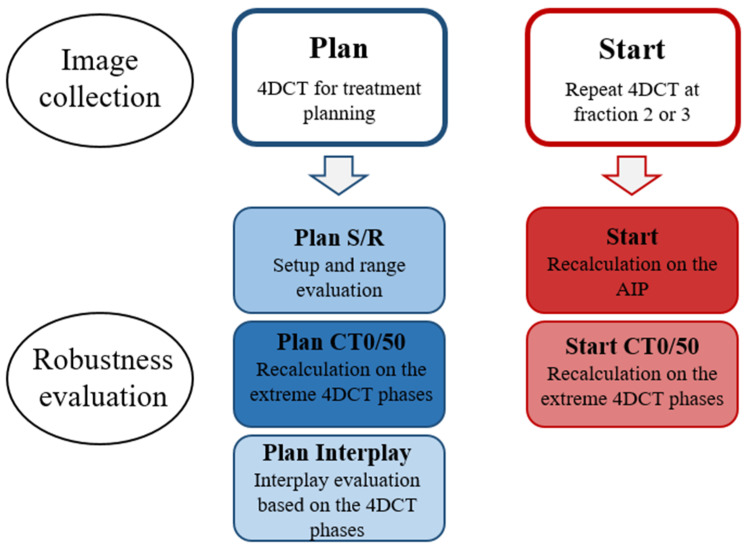
Overview of the acquired image data and the robustness evaluation.

An overview of the acquired image data and the robustness evaluation is shown in Figure 1. Robustness towards *setup and range* variations (Plan S/R) was evaluated on the Plan AIP using combined isocenter shifts of 2.9 mm in 3 directions simultaneously (corresponding to 5 mm isotropic shifts) and 3.5% range uncertainty (16 scenarios).

Robustness towards *breathing motion* at Plan (Plan CT0/50) was evaluated by recalculating all PT plans on the extreme breathing phases of the 4DCT: CT0 (maximum inspiration) and CT50 (maximum expiration). In addition, *interplay* evaluation was performed at Plan (Plan Interplay) using a script provided by RaySearch. The 10 breathing phases of the 4DCT were in turn used as the starting phase for treatment delivery, and the spots were distributed on the CTs of the different phases based on delivery time and breathing cycle length. Constant breathing periods of five seconds were used for all patients. The dose on each phase was calculated and mapped to the reference image (AIP), where the total dose was calculated. This resulted in 10 different interplay dose distributions depending on which phase delivery started in. For all robustness simulations, reported values represent the worst-case scenario for each parameter.

All PT plans were also recalculated on the AIP (Start), CT0 and CT50 (Start CT0/50) of the Start 4DCT to evaluate robustness towards *changes in breathing motion* and *anatomy* that can occur between planning and onset of treatment.

### 2.4. Dosimetric Evaluation

Dose distributions at Plan were compared using D_98%_ and D_2%_ for the CTV, as well as the homogeneity index HI = (D_2%_ − D_98%_)/D_50%_, and the conformity index CI = (TV_RI_/TV) × (TV_RI_/V_RI_), where TV is the target volume, TV_RI_ is the target volume covered by the reference (95%) isodose and V_RI_ is the volume of the reference isodose [30]. For healthy tissue and OARs, the following parameters relevant for toxicity were evaluated: D_2cc_ for the patient body, D_mean_, V_5Gy_ and V_20Gy_ for the lungs, D_mean_ and V_30Gy_ for the heart, D_mean_ for the esophagus and D_max_ for the spinal canal.

For OARs, the planning criteria (Table A1) were also required in robustness evaluation. In addition, the D_2cc_ to the patient body should be <107% of the prescribed dose. CTV D_98_ > 95% and CTV D_2%_ < 107% were required in setup and range and extreme phase evaluation as well as in the Start recalculations. The interplay effect is expected to cause under- and overdosage in the tumor and OARs that average out during fractionated treatment. Ensuring at least 1.8 Gy per fraction, i.e., CTV D_98_ > 90%, and CTV D_2%_ and body D_2cc_ < 110% were considered acceptable in interplay evaluation.

A structured overview of the various evaluations and criteria is shown in Table A2. Initially, we present the target coverage and OAR sparing for the various techniques at Plan. Thereafter, we investigate the robustness of the target dose and OAR doses, respectively. For evaluation of the actual clinical potential of proton therapy compared to photon therapy, we lastly compare target coverage and OAR sparing at Start.

### 2.5. Statistical Analysis

Statistical analyses were performed in IBM SPSS Statistics (IBM Corp., Armonk, NY, USA). Friedman’s test (non-parametric two-way analysis of variance by ranks) was used for comparison of the different techniques. Bonferroni correction was applied to adjust the *p*-value for multiple testing in post hoc analysis. A significance level of 0.05 was used.

## 3. Results

### 3.1. Patient Characteristics and Breathing Motion

The median CTV volume was 137 cc (range 66–435 cc). Nine patients had disease stage IIIA, five IIIB and one IIIC. Primary tumor positions were left upper (3), left lower (4), right upper (4) and right lower (3) lobe. One patient only had mediastinal lymph nodes and one only had a primary tumor; the rest had both primary tumor and lymph nodes included in the target volume. The prescribed dose was 60 Gy for 6 patients and 66 Gy for 9 patients.

The breathing motion of the primary tumor was largest in the cranio–caudal direction, with a median amplitude of 4 mm and a maximum of 15 mm in the planning 4DCTs. Large variability in breathing motion was observed between patients. Six patients had a motion amplitude >5 mm, all in the cranio–caudal direction. Median breathing motion amplitudes were similar at Plan and Start (Table A3), and for most patients, the change in amplitude from Plan to Start was ≤2 mm in all directions. Three patients had a larger change in amplitude in the cranio–caudal direction (−6 mm, +4 mm and −3 mm), and these were also the three patients with the largest breathing motion amplitudes at Plan.

### 3.2. Target Coverage and OAR Sparing at Plan

**Figure 2:**
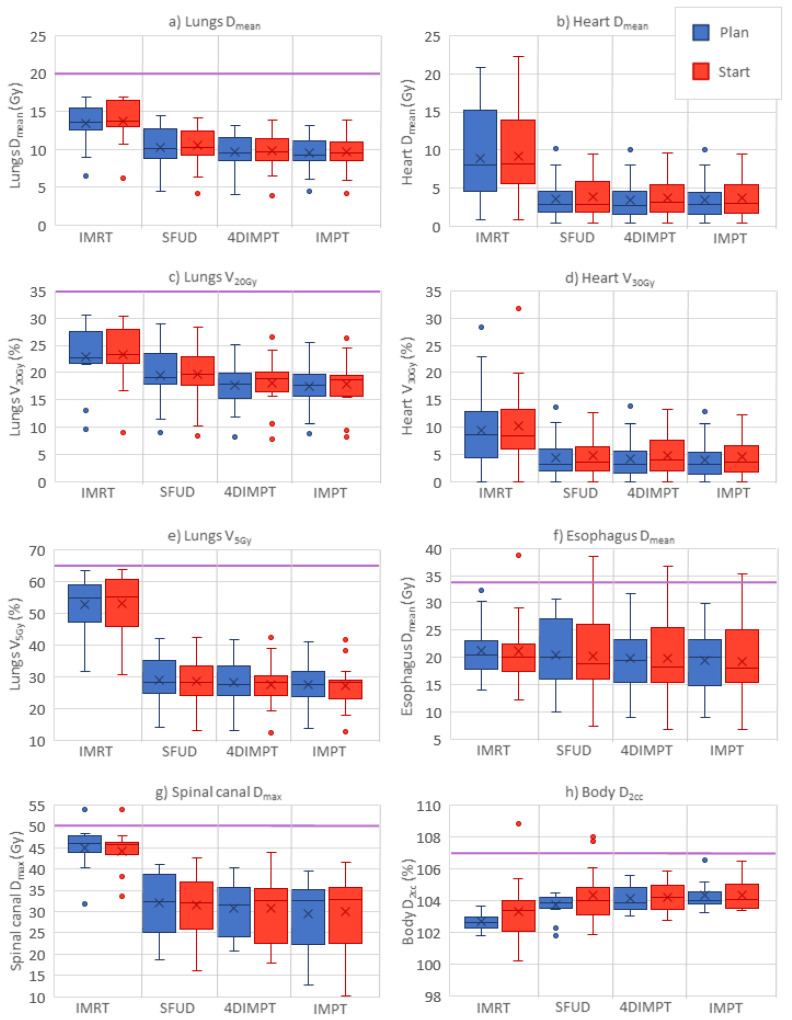
OAR and normal tissue doses at Plan (blue) and Start (red) for the different optimization techniques; panel (**a**–**h**) shows each of the evaluated parameters. Purple horizontal lines indicate planning constraints in cases where the value is included in the plot. Boxplots show the median (line), mean (cross) and spread, with outliers as dots outside the box. IMRT = intensity-modulated radiotherapy, SFUD = single-field uniform dose, 4DIMPT = 4D robustly optimized intensity-modulated proton therapy, IMPT = 3D robustly optimized intensity-modulated proton therapy.

All treatment plans achieved the required CTV D_98%_ > 95% and D_2%_ < 107% of the prescribed dose at Plan (Table 1). The median CTV D_98%_ was, however, significantly higher for IMRT than for all PT techniques. The median PTV D_98%_ in the IMRT plans was 95.7% (range 94.6–97.0%). Healthy tissue and OAR doses were lower for all proton techniques than for IMRT (Table 1, Figure 2). The only exception was the D_2cc_ of the body, where IMRT gave the lowest dose. Among the PT techniques, significant differences were found between SFUD and IMPT in D_mean_ for the lungs and esophagus and V_20Gy_ for the lungs, all in favor of IMPT. The mean rank was the worst with IMRT and the best with IMPT for all of the evaluated OAR parameters.

### 3.3. Target Dose Robustness at Plan and Start

**Figure 3:**
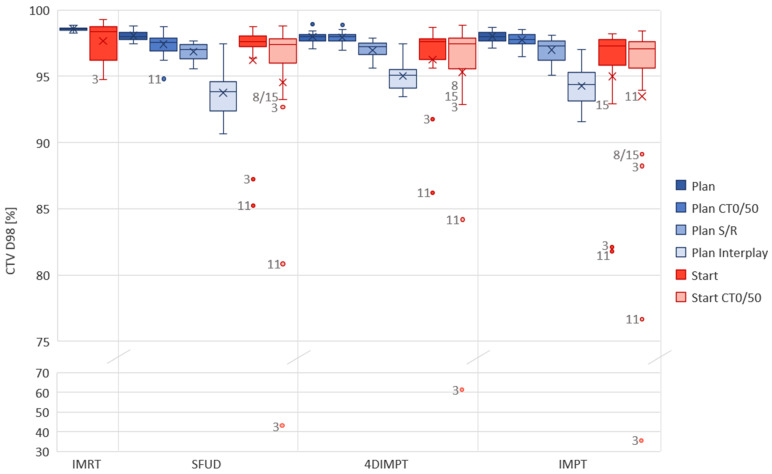
Robust evaluation on the Plan (blue) and Start (red) CTs for each optimization technique. The acceptance criteria were D_98%_ > 90% for interplay evaluation and >95% for other robust evaluations. In cases where the criteria were not met, the patient number is given next to the observation in the figure. DIBH CT was used in IMRT for patients 3 and 11, hence planning and recalculation were not performed on the same scans as for the PT plans. CT0/50 includes two observations (both extreme phases) per patient. Boxplots show the median (line), mean (cross) and spread, with outliers as dots outside the box. Plan = planning CT, Start = start of treatment CT, CT0/50 = extreme phase evaluation, S/R = setup and range evaluation.

All IMPT and 4DIMPT plans achieved the criteria for D_98%_ and D_2%_ for the CTV on setup and range evaluation and extreme phase evaluation at Plan (Figure 3). One SFUD plan narrowly failed with a D_98%_ of 94.8% on CT0. In interplay evaluations, all plans fulfilled the goal of D_98%_ > 90%. CTV D_2%_ slightly exceeded 107% in interplay evaluations of three plans, two of which were SFUD and one was 4DIMPT. Thus, all PBS-PT techniques had satisfying target robustness at Plan. The results from the extensive robustness evaluation at Plan and Start for CTV D_98%_ are shown in Figure 3, and a summary of D_98%_ and D_2%_ values for all evaluations of all proton techniques are listed in the Appendix A (Table A4).

For the Start AIP recalculation, the CTV D_98%_ was above 95% of the prescribed dose for 13/15 patients with SFUD and 4DIMPT and 12/15 with IMPT (Figure 3). In general, the CTV D_98%_ decreased in extreme-phase evaluations, but for 11/15 patients, it was still above 95%, independent of the optimization technique. The differences in the median CTV D_98%_ between the PT techniques were small for both AIP and CT0/50 recalculations at Start (Figure 3, Table A4). It was, however, statistically significant between 4DIMPT and IMPT on CT0/50, in disfavor of IMPT. D_2%_ was similar and <107% for all plans on all scans.

**Figure A2:**
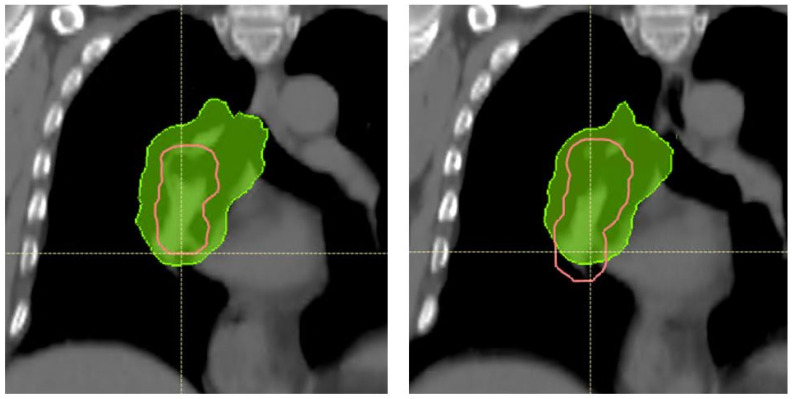
SFUD plan for patient 3. Deeper breathing and increased breathing motion causes the CTV (pink) in the mediastinum to extend 15 mm more caudally and 3 mm more cranially on the Start AIP (**right**) compared to the Plan AIP (**left**). The yellow cross shows a reference position in both images. The 95% isodose (green) does not cover the caudal area of the CTV at Start. Notably, the diaphragm position had also changed in this patient, and this change would be detectable on CBCT imaging.

**Figure A3:**
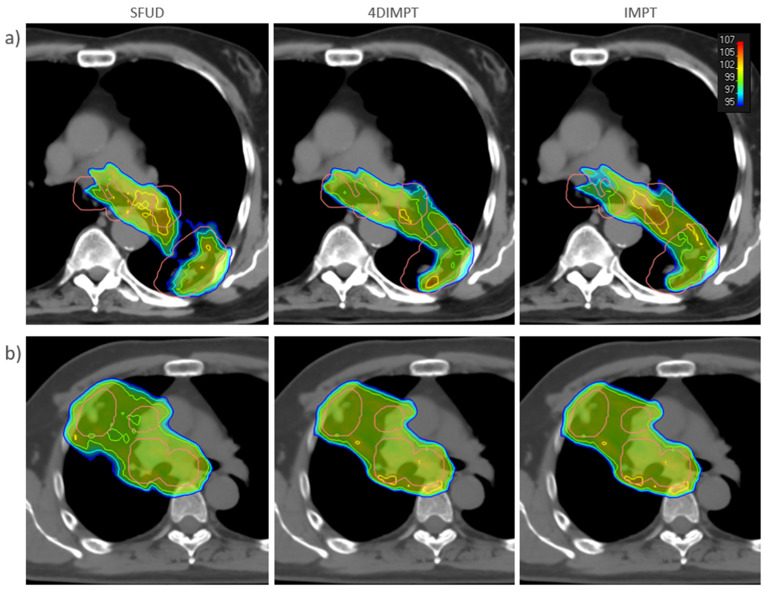
SFUD (**left**), 4DIMPT (**middle**), and IMPT (**right**) plans recalculated on the Start AIP for (**a**) one of the patients that failed the robustness criteria (patient 11) and (**b**) one of the patients with satisfactory target coverage (patient 10). The CTV is delineated in pink. Doses higher than 95% of the prescribed dose are shown, according to the color scale in the top right corner.

One of the patients that stood out with insufficient CTV coverage at Start (AIP) was patient 3, with D_98%_ of 87.2%, 91.7% and 82.7% for SFUD, 4DIMPT and IMPT, respectively. This patient had a change in breathing pattern between Plan and Start, causing the CTV in the mediastinum to expand 15 mm caudally and 3 mm cranially (Figure A2). Similar changes were seen for patients 11 and 15. The CTV coverage at Start for these patients was not sufficient with any PT optimization technique (Figure 3). For one of the patients with insufficient and one of the patients with sufficient CTV coverage at Start, dose distributions for all PT techniques are shown in Figure A3. For patient 8, the low CTV D_98%_ at the Start CT0/50 was likely caused by delineation uncertainty. The IMRT plans for patients 3 and 11 were planned and recalculated on DIBH CTs. The values for IMRT and PT techniques can therefore not be directly compared.

### 3.4. OAR Dose Robustness at Plan and Start

In setup and range and extreme-phase evaluations at Plan, both IMPT and 4DIMPT achieved the constraints for OARs (Table A1) in all plans. One of the SFUD plans failed in the setup and range evaluation, exceeding the D_max_ criterion for the spinal canal with 52.0 Gy in the worst-case scenario. In interplay evaluation, 26 out of 45 plans had a D_2cc_ to the body >107%; however, only three plans exceeded 110% of the prescribed dose. Two of these were IMPT plans, and one was 4DIMPT. The OAR constraints were met for all patients and all techniques in interplay evaluation.

Relevant dose-volume parameters for OARs at Plan and Start for IMRT and all PT techniques are shown in Figure 2. The pattern of OAR sparing with PT compared to IMRT persisted at Start. Median changes in dose-volume parameters from Plan to Start were 6% or lower for all parameters and all techniques (Table A5). Nevertheless, large variations between patients in the relative change of dose-volume parameters (ranging from −58% to 103%) from Plan to Start were seen for individual patients with all techniques. For most of the patients, constraints were still achieved for all OARs. For one patient, the esophagus shifted towards the CTV, causing a ~30% increase in mean dose to above 35 Gy for all techniques. Hotspots (D_2cc_ > 107%) to the healthy tissue occurred at Start with one IMRT plan and two SFUD plans.

### 3.5. Target Coverage and OAR Sparing at Start

**Figure 4:**
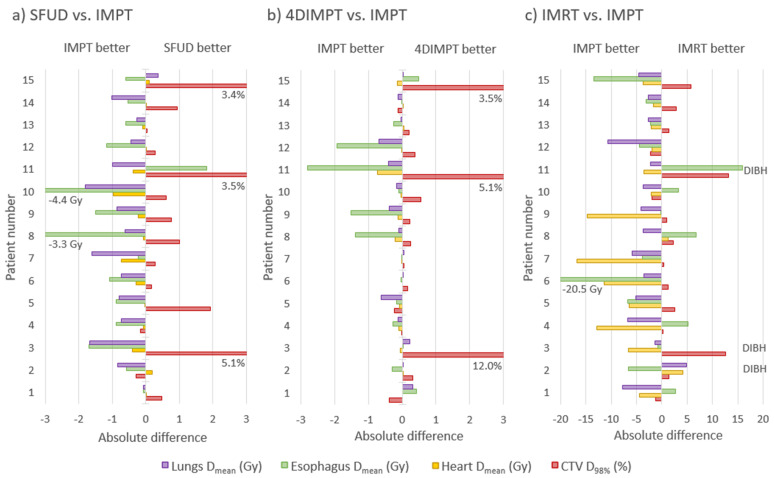
Absolute difference in mean OAR dose and CTV D_98%_ per patient between IMPT and SFUD (**a**), IMPT and 4DIMPT (**b**) and IMPT and IMRT (**c**) as calculated on the AIP at Start. Negative values are always in favor of IMPT (for the OARs, the other technique is subtracted from IMPT, while for the CTV, IMPT is subtracted from the other technique). The patients are sorted according to increasing breathing motion. Patient 1 had no primary tumor, patients 2–7 had tumor motion amplitude >0.5 cm and patients 8–15 had tumor motion amplitude <0.5 cm. Notably, the patients with a large advantage in CTV D_98%_ for SFUD and 4DIMPT (patients 3, 11 and 15) are the same patients where changes in breathing pattern and anatomy deteriorated CTV coverage for all proton techniques. Note also that three of the patients (2, 3 and 11) received IMRT in DIBH and cannot be directly compared in (**c**).

Out of the PBS-PT techniques, IMPT showed the greatest potential for toxicity reduction. A comparison of all 105 OAR dose-volume parameters calculated at Start resulted in the best mean rank for IMPT (1.51), followed by 4DIMPT (2.06), SFUD (2.56) and IMRT (3.87), with all pairwise comparisons being significant. Figure 4 shows the per-patient advantage of IMPT in the sparing of OAR mean doses, as well as the price to pay in target coverage. The latter was, however, only significantly different between IMPT and IMRT, probably influenced by the use of DIBH for IMRT.

**Figure 5:**
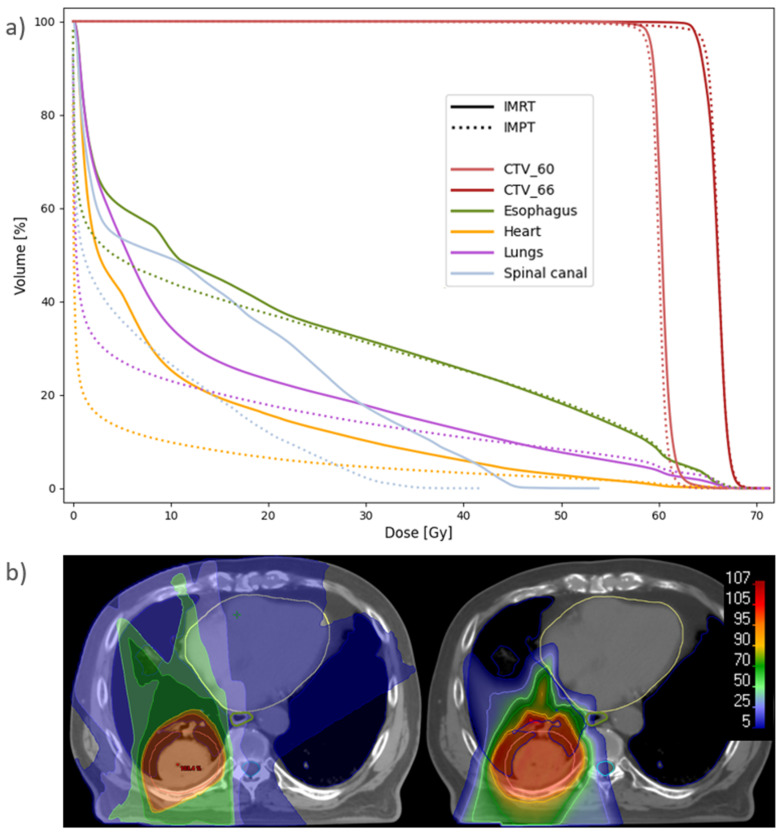
(**a**) Population average DVH for IMRT and IMPT plans recalculated at Start. Six patients had CTV_60 and nine had CTV_66. (**b**) Dose distribution for patient 7 with IMRT (**left**) and IMPT (**right**), recalculated at Start. The spread of the low and medium doses results in higher mean doses to the lungs (blue), esophagus (green) and heart (yellow) with IMRT. The CTV is delineated in pink, and the spinal canal in cyan.

Substantial dose reductions were achieved with IMPT compared to state-of-the-art IMRT for the lungs, heart and spinal canal (Figure 4 and Figure 5). For the lungs, the median D_mean_ was reduced from 13.7 to 9.6 Gy, V_5Gy_ from 55.1 to 28.4% and V_20Gy_ from 23.4 to 18.6% with IMPT compared to IMRT. The median heart D_mean_ was reduced from 8.2 to 3.0 Gy, with D_mean_ < 10 Gy for all patients with IMPT and 10/15 with IMRT. The median heart V_30Gy_ was reduced from 8.3 to 3.6%, the median esophagus D_mean_ was reduced from 20.1 to 18.1 Gy and the median spinal canal D_max_ was reduced from 45.5 to 32.7 Gy. All differences were statistically significant, and among the 105 individual parameters compared, 102 were in favor of IMPT, 2 were in favor of IMRT, and 1 was tied.

## 4. Discussion

This study shows that the potential for OAR sparing with PBS-PT compared to state-of-the-art IMRT was substantial and persistent from the planning to the start of treatment. Among the various optimization techniques, IMPT spared OARs the most. There were surprisingly small differences between the PBS-PT techniques in the response to various uncertainties, but IMPT was slightly less robust towards breathing motion than 4DIMPT. All techniques were acceptable with respect to robustness evaluations at Plan, including interplay, and also at Start for the majority of patients. However, all robust optimization techniques failed to account for changes in breathing motion patterns occurring in three patients, causing unacceptable coverage of the mediastinal lymph nodes.

Given strategies to recognize patients with altered breathing motion and account for the lack of target robustness in these patients, we believe robustly optimized IMPT and 4DIMPT can reduce the risk of both radiation pneumonitis and heart toxicity compared to IMRT. Lung D_mean_, V_5Gy_ and V_20Gy_ were all significantly reduced with IMPT and 4DIMPT, and these parameters have previously been correlated to the probability of radiation pneumonitis [31]. Interestingly, this could potentially be a key to better outcome as well, since patients with radiation pneumonitis have been excluded from adjuvant treatment with immune checkpoint inhibitors [32]. Note that SFUD did not reduce lung doses compared to IMRT in our study, and therefore we would not expect any reduced risk of pneumonitis with SFUD. The reductions in heart dose seen with all PBS-PT techniques are also likely clinically relevant. Atkins et al. showed that a heart D_mean_ > 10 Gy significantly increased the risk of mortality in LA-NSCLC [33]. In our study, a mean dose to the heart below 10 Gy was achieved for 10/15 patients with IMRT and for all patients across all PBS-PT techniques.

Sparing of the spinal canal beyond the max dose constraint is not expected to give a clinical benefit in itself. However, with the large reduction seen with all PBS-PT techniques compared to IMRT, less effort must be spent on this highly prioritized constraint in the optimization, possibly giving room for the considerable dose reduction seen for other OARs.

Mean doses to the esophagus were slightly reduced with IMPT compared to IMRT in our study. It is unknown whether this would lead to a reduction in esophagitis [34], especially since there are additional uncertainties in elevated LET that were not considered in the current study. The esophagus is highly mobile and often located in close proximity to the target volume, and can move into the high-dose region. This was the case for one of the patients in our study. In a recent clinical dose-escalation study (including 47 patients with stage III NSCLC) by Iwata et al., ART was used to monitor the position of the esophagus and adjust treatment accordingly if needed [8]. Dose-escalated PT was well tolerated in this phase II study, with no grade ≥3 radiation pneumonitis and one case of acute grade 3 esophagitis. Additionally, the 5-year overall survival of 59% (probably influenced by combination with immunotherapy) shows promise. This study mainly used passive scattering PT, although some patients with small tumor motion had single-field optimized spot-scanning plans.

Recently, Ribeiro et al. published a comprehensive robustness analysis, including weekly imaging during treatment, for 10 stage III NSCLC patients with small to moderate tumor motion, showing the feasibility of PBS-PT in the majority of patients [27]. Our study strengthens these findings by confirming the results in an independent patient group with larger motion variability. Inoue et al. also investigated the robustness of 3D robustly optimized IMPT in stage III NSCLC [35]. They reported a limited impact of setup and range uncertainties, breathing motion and interplay effects on the dose distribution when using properly selected robust optimization parameters. This is in line with our analysis for the planning scan.

A strength of our study was the prospective study design with repeated imaging at the start of treatment. At this time point, we expected a small probability of anatomical changes in need of ART, based on experience from photon therapy [36]. A CT at fraction 2 or 3 was therefore chosen for robustness evaluation, as it would reveal if any of the optimization techniques were particularly sensitive towards interfractional variations such as changes in breathing pattern or positioning of the patient.

Indeed our results show that none of the optimization techniques for PBS-PT were able to handle substantial changes in the breathing pattern. With current robust optimization methods, it is therefore important to verify dose delivery at the onset of treatment. Adaptive protocols in PT are commonly based on weekly 4DCTs, starting at the end of the first treatment week, but imaging at the onset of treatment could recognize these patients earlier. Importantly, the observed target under-dosage was mainly located in the mediastinal lymph nodes (and not the primary tumor), which are hard to locate on, e.g., CBCT. A possibility is to use the carina as a surrogate structure in addition to the diaphragm, as done by Møller et al. in their ART protocol [36]. The carina position has been shown to correlate better with lung volume than, e.g., diaphragm position [37]. Alternative strategies to avoid dose degradation due to breathing motion changes could be respiratory gating or breath-hold strategies. Although images in DIBH were acquired in the current study and used clinically in IMRT for three patients, analysis of PBS-PT in DIBH was beyond the scope of the current study.

Both Ribeiro et al. and Hoffmann et al. reported that altered shoulder position caused a loss in robustness when evaluating dose during treatment [27,28]. This was not observed in our study, but in principle, this could also occur from the planning to the start of treatment and should be kept in mind when evaluating robustness at the onset of treatment. Our study design was limited to observing changes between the planning and start of treatment, and hence anatomical changes such as atelectasis or pleural effusion were not observed. Such changes can occur during treatment and largely impact the delivered dose, but they are well known and can be corrected for by existing adaptive protocols [28]. The novelty of our study lies in focusing on uncertainties that so far have received less attention. We have shown that these are neither handled by current robust optimization techniques nor adaptive protocols.

Regarding the comparison of different PBS-PT optimization techniques, Ribeiro et al. compared 3D and 4D robustly optimized IMPT plans with layered rescanning in their study [27]. Similar to us, they found only small differences in robustness between the techniques. However, IMPT was (somewhat surprisingly) slightly more robust than 4DIMPT in their study, while we found the opposite. This might be explained by the difference in the use of density override for the target. In the study by Ribeiro et al., density override was only used for the IMPT plans, while we used it on the AIP for both techniques. This is an example of one out of several technical details that might influence robustness; rescanning is another [14]. Indeed, with the use of rescanning, the uncertainties due to the interplay effect could be limited even further than reported here. Liao and colleagues have pointed out the importance of treatment planning experience in PT for NSCLC [11]. In addition to comprehensive treatment planning guidelines, solutions for automated treatment planning could be useful to ensure the high plan quality needed in PBS-PT for LA-NSCLC [38].

The number of treatment fields could also influence the robustness of the PT plans. In this study, two fields were used for ten patients and three fields for five patients. On the one hand, adding a third field could increase the robustness, as the dose contribution is divided between more treatment angles, and changes in anatomy affecting one of the fields have a lesser impact on the dose distribution. However, some issues came with increasing the number of fields. For some patients, finding a third, robust angle could be difficult due to, e.g., arm position or large breasts or fat folds, where it was preferred to avoid beam entry due to positioning uncertainty. In the 4D optimization, splitting the fields with field-specific targets was not possible, so the fields had to be able to contribute to both the primary tumor and the lymph node volumes, giving some limitations for robust angles because of the surrounding anatomy. Hence, the requirement for the field setup to work for all optimization techniques was a limitation in this study.

Another limitation of the current study is the low number of included patients. Despite this, there was a large variation in tumor size and position, and breathing motion ranged from negligible to substantial. These parameters also varied among the patients that failed the robustness criteria at Start. Finally, the 4D optimization and extreme phase and interplay evaluations performed in this study required deformable image registration and mapping of contours to each phase of the 4DCT. As delineation was performed on the AIP as a part of the clinical routine, the contours were mapped from the AIP to each phase. Due to blurring of the edges, the GTV on the AIP may be slightly larger than in reality, and the plans may therefore be slightly more robust than if delineation had been performed on one of the phase images.

## 5. Conclusions

The potential of IMPT and 4DIMPT for reducing heart and lung toxicity in the treatment of LA-NSCLC was substantial and persistent at Start. SFUD only showed potential for reduced heart toxicity. All proton optimization techniques responded similarly to uncertainties and were sufficiently robust towards setup and range uncertainties as well as interplay at Plan, and for the majority of patients in recalculations at Start. Altered breathing patterns between Plan and Start jeopardized target coverage for all PBS-PT techniques. Adaptive protocols for free-breathing PBS-PT should include imaging at onset of or early in treatment, and possibly a surrogate for visualization of the mediastinal target. Given such strategies to recognize patients with altered breathing patterns, we believe there is great potential for PBS-PT to improve the treatment of LA-NSCLC.

## Figures and Tables

**Table 1:** Dose-volume parameters for the target, patient body, and OARs for the different optimization techniques, evaluated on the planning scan. The mean rank (obtained by Friedman’s test) for each technique regarding each evaluated parameter is also shown. A mean rank of 1 would mean that this was the best plan for all patients, while a mean rank of 4 means that this was the worst plan for all patients. Statistically significant differences (*p* < 0.05) compared to the IMRT plan are shown in bold.

	IMRT		SFUD		4DIMPT		IMPT	
	Median (Range)	Mean Rank	Median (Range)	Mean Rank	Median (Range)	Mean Rank	Median (Range)	Mean Rank
CTV D_98%_ (%)	98.6 (98.2–98.8)	1.20	**98 (97.4–98.8)**	2.93	**98 (97.1–98.9)**	2.93	**98 (97.1–98.7)**	2.93
CTV D_2%_ (%)	102.2 (101.7–102.7)	1.93	102.5 (101.5–103.0)	2.13	102.6 (102–103.4)	2.93	102.4 (102.2–103.9)	3.00
CTV CI	0.41 (0.33–0.62)	1.70	**0.34 (0.26–0.53)**	3.33	0.34 (0.25–0.64)	2.50	0.34 (0.26–0.61)	2.47
CTV HI	0.036 (0.030–0.044)	1.27	**0.046 (0.027–0.051)**	2.53	**0.047 (0.038–0.059)**	3.13	**0.044 (0.036–0.068)**	3.07
Body D_2cc_ (Gy)	67.5 (61.3–68.4)	1.20	**68.3 (61.1–69.0)**	2.47	**68.1 (62.1–69.7)**	3.07	**68.5 (61.9–69.4)**	3.27
Lungs D_mean_ (Gy)	13.6 (6.6–16.8)	3.93	10.2 (4.5–14.5) *	2.87	**9.6 (4.1–13.2)**	1.87	**9.2 (4.5–13.1)**	1.33
Lungs V_5Gy_ (%)	54.9 (31.8–63.2)	4.00	**28.3 (14.0–41.9)**	2.47	**27.5 (13.1–41.6)**	2.00	**27.6 (13.7–40.9)**	1.53
Lungs V_20Gy_ (%)	22.6 (9.6–30.6)	3.80	19.0 (9.0–29.0) *	2.93	**18.0 (8.2–25.1)**	1.93	**17.6 (8.8–25.5)**	1.33
Heart D_mean_ (Gy)	8.1 (0.9- 20.7)	4.00	**2.8 (0.5–10.1)**	2.53	**2.8 (0.5–10.0)**	1.93	**2.8 (0.5–10.0)**	1.53
Heart V_30Gy_ (%)	8.6 (0.0–28.2)	3.90	**3.2 (0.0–13.7)**	2.30	**3.3 (0.9–13.9)**	2.37	**3.3 (0.0–13.7)**	1.43
Esophagus D_mean_ (Gy)	20.4 (14.1–32.2)	3.33	20.0 (10.0–30.8) *	3.00	19.5 (8.9–31.8)	2.27	**20.0 (9.0–29.9)**	1.40
Spinal Canal D_max_ (Gy)	46.0 (31.8–53.9)	4.00	**32.3 (18.8–41.1)**	2.40	**31.4 (20.6–40.3)**	2.00	**32.5 (12.8–39.6)**	1.60

* Statistically significant difference between SFUD and IMPT.

## Data Availability

The data presented in this study are available on request from the corresponding author. The data are not publicly available due to privacy reasons as they are part of an ongoing study.

## Appendix A

**Table A1:** Planning dose constraints for organs at risk.

Organ	Dose Constraint
Lungs	V_5Gy_ < 65% V_20Gy_ < 35% D_mean_ < 20 Gy
Esophagus	D_mean_ < 34 Gy
Heart	V_30Gy_ < 40%
Spinal canal	D_max_ < 50 Gy
Brachial plexus	D_max_ < 66 Gy

**Table A2:** Overview of the performed robustness evaluations, and the criteria used in each evaluation. The criteria in parentheses were used for interplay evaluation. * OAR constraints are shown in Table A1.

Evaluations of	Evaluations at Plan	Evaluations at Start	Evaluation Criteria
Target coverage and OAR sparing at Plan	Plan AIP		CTV D_98%_ > 95% CTV D_2%_ < 107% Body D_2cc_ < 107% OARs within constraints *
Target dose robustness	Plan S/R Plan CT0/50 Plan Interplay	Start AIP Start CT0/50	CTV D_98%_ > 95% (90%) CTV D_2%_ < 107% (110%)
OAR dose robustness	Plan S/R Plan CT0/50 Plan Interplay	Start AIP Start CT0/50	Body D_2cc_ < 107% (110%) OARs within constraints *
Target coverage and OAR sparing at Start		Start AIP	CTV D_98%_ > 95% CTV D_2%_ < 107% Body D_2cc_ < 107% OARs within constraints *

**Table A3:** Breathing motion amplitudes of the primary tumor in the Plan and Start 4DCTs. Median value and range are given for each direction.

Direction	Motion—Plan	Motion—Start
x (left–right)	1 mm (0–5)	1 mm (0–6)
y (anterior–posterior)	2 mm (1–4)	2 mm (1–4)
z (cranio–caudal)	4 mm (1–15)	4 mm (1–13)

**Table A4:** Robust evaluation of CTV dose, showing median values and range.

Parameter	Evaluation	SFUD	4DIMPT	IMPT
CTV D_98%_ (%)	Plan CT0/50	97.5 (94.8–98.8)	98.0 (97.0–99.0)	97.8 (96.5–98.5)
	Plan setup/range	97.0 (95.6–97.7)	97.2 (95.6–97.7)	97.3 (95.1–98.1)
	Plan interplay	93.6 (90.7–97.5)	95.0 (93.5–97.0)	94.6 (91.8–97.0)
	Start	97.6 (85.3–98.8)	97.6 (86.2–98.7)	97.5 (81.8–98.2)
	Start CT0/50	97.4 (43.2–98.8)	97.4 (61.3–98.9)	97.1 (35.6–98.4)
CTV D_2%_ (%)	Plan CT0/50	102.2 (101.2–103.2)	102.5 (102.0–103.5)	102.5 (102.0–103.9)
	Plan setup/range	103.3 (102.2–104.5)	103.2 (102.3–104.2)	103.3 (102.5–105.5)
	Plan interplay	104.9 (102.7–107.6)	105.3 (103.2–107.5)	105.5 (104.1–106.7)
	Start	102.6 (101.4–104.4)	102.8 (102.0–103.7)	102.6 (101.8–103.7)
	Start CT0/50	102.3 (101.1–104.9)	102.5 (102.0–104.0)	102.5 (101.8–103.8)

**Table A5:** Difference in OAR parameters from Plan to Start, relative to the Plan value. Median and range are shown for each technique. Positive values indicate a higher dose and negative values indicate a lower dose at Start.

Structure	Difference, Plan vs. Start			
	IMRT	SFUD	4DIMPT	IMPT
Lungs D_mean_ (Gy)	1 (−6/20)	0 (−11/20)	0 (−11/33)	0 (−12/32)
Lungs V_5Gy_ (%)	−1 (−5/7)	−1 (−11/26)	−2 (−11/26)	−1 (−12/28)
Lungs V_20Gy_ (%)	1 (−7/29)	3 (−13/30)	2 (−10/30)	3 (−11/31)
Heart D_mean_ (Gy)	−4 (−13/68)	−6 (−55/70)	−4 (−47/78)	−6 (−51/78)
Heart V_30Gy_ (%)	0 (−29/162)	0 (−58/84)	0 (−42/97)	0 (−50/103)
Esophagus D_mean_ (Gy)	−3 (−17/28)	−5 (−27/30)	−4 (−31/31)	−4 (−31/35)
Spinal canal D_max_ (Gy)	−1 (−9/6)	1 (−14/6)	0 (−16/9)	1 (−21/8)
Body D_2cc_ (Gy)	0 (−2/6)	0 (−1/4)	0 (0/1)	0 (−3/1)
